# Integrin Expression in Esophageal Squamous Cell Carcinoma: Loss of the Physiological Integrin Expression Pattern Correlates with Disease Progression

**DOI:** 10.1371/journal.pone.0109026

**Published:** 2014-11-14

**Authors:** Christian Vay, Stefan B. Hosch, Nikolas H. Stoecklein, Christoph A. Klein, Daniel Vallböhmer, Björn-Christian Link, Emre F. Yekebas, Jakob R. Izbicki, Wolfram T. Knoefel, Peter Scheunemann

**Affiliations:** 1 Department of Surgery (A), Heinrich-Heine-University and University Hospital Düsseldorf, Düsseldorf, Germany; 2 Department of General, Visceral, and Thoracic Surgery, University Medical Center Hamburg-Eppendorf, Hamburg, Germany; 3 Department of General, Vascular, and Visceral Surgery, Ingolstadt Medical Center, Ingolstadt, Germany; 4 Division of Oncogenomics, Institute of Pathology, University of Regensburg, Regensburg, Germany; 5 Department of Pediatric Surgery, University Hospital Rostock, Rostock, Germany; Thomas Jefferson University, United States of America

## Abstract

The integrins are a family of heterodimeric transmembrane signaling receptors that mediate the adhesive properties of epithelial cells affecting cell growth and differentiation. In many epithelial malignancies, altered integrin expression is associated with tumor progression and often correlates with unfavorable prognosis. However, only few studies have investigated the role of integrin expression in esophageal squamous cell carcinoma (ESCC). Using a novel quantifying immunofluorescence-staining assay, we investigated the expression of the integrins α_2_β_1_, α_3_β_1_, α_6_β_1_, and α_6_β_4_ in primary ESCC of 36 patients who underwent surgical resection. Magnitude and distribution of expression were analyzed in primary tumor samples and autologous esophageal squamous epithelium. The persistence of the physiologically polarized expression of the subunits α_6_, β_1_, and β_4_ in the tumor tissue was significantly associated with prolonged relapse-free survival (p = 0.028, p = 0.034, p = 0.006). In contrast, patients with reduced focal α_6_ expression at the tumor invasion front shared a significantly shortened relapse-free survival compared to patients with strong α_6_ expression at their stromal surfaces, as it was regularly observed in normal esophageal epithelium (p = 0.001). Multivariate regression analysis identified the maintenance of strong α_6_ immunoreactivity at the invasion front as an independent prognostic factor for increased relapse-free and disease-specific survival (p = 0.003; p = 0.003). Our findings suggest that alterations in both pattern and magnitude of integrin expression may play a major role in the disease progression of ESCC patients. Particularly, the distinct expression of the integrins α_6_β_4_ and α_6_β_1_ at the invasion front as well as the maintenance of a polarized integrin expression pattern in the tumor tissue may serve as valuable new markers to assess the aggressiveness of ESCC.

## Introduction

Esophageal cancer is a highly aggressive tumor entity characterized by late diagnosis and early metastasis [1], [2]. As the eighth most common cancer worldwide with over 480,000 new cases estimated in 2008, and the sixth most common cause of death from cancer worldwide with 407,000 deaths (5.4% of the total) in 2008, esophageal carcinoma is one of the leading causes of malignancy-associated death [3], [4]. Even though the incidence of esophageal adenocarcinoma has been rising in most western industrial countries like no other malignancy since the mid-1970s, globally squamous cell carcinoma (SCC) still represents a predominant type of esophageal cancer and accounts for the a high number of fatal outcomes [5]. Due to advances in surgical techniques and multimodal treatment strategies, the prognosis of esophageal cancer has improved over the last two decades [6]–[8]. However, survival rates remain unsatisfactory and continue to lag behind those of other gastrointestinal malignancies [9]. At present, postoperative clinicopathological staging is still the most relevant factor to estimate disease recurrence and patient survival [10].

The integrins are a family of ubiquitously expressed transmembrane glycoprotein receptors composed of non-covalently linked α and β polypeptide subunits [11]. Integrins mediate cell-to-cell and cell-to-extracellular matrix adhesive interactions and transduce signals from the extracellular matrix (ECM) to the cell interior and vice versa [12], [13]. The intracellular domains of the integrin subunits link the cell surface to the actin and myosin cytoskeleton by adaptor proteins influencing cellular structure and motility [14]. Furthermore, these domains relay integrin receptor signaling, which – in concert with growth factor receptor downstream signaling – significantly influences cell cycle progression, differentiation and survival [15]. Aside from their pivotal functions in embryonic development and tissue organization, these properties determine a key role for the integrins in the formation and progression of malignant tumors in general [14], [16], [17], and squamous cell carcinomas in particular [18].

Comparing the overall distribution of integrins in epithelial malignancies to the physiological expression in the non-malignant tissue of origin, especially the laminin-binding integrins α_2_β_1_, α_3_β_1_, α_6_β_1_, and α_6_β_4_ exhibit an aberrant expression behavior in a broad range of carcinomas [19]–[31]. Infiltrative growth of malignant epithelial tumors is initiated by the penetration of the basement membrane (BM), which also serves as storage for growth factors, cytokines, and other mediators [32], [33]. Along with collagen, nidogen, and proteoglycans, the laminins – a family of trimeric ECM glycoproteins – are a major component of the BM. The ligation of laminin by integrin receptors offers structural support to the adjacent epithelial cell layer and triggers integrin “outside-in” signaling, which strongly influences the proliferative behavior of basal keratinocytes and their subsequent differentiation in squamous epithelia [34]. Thus, the laminin-binding integrins contribute to the maintenance of the structural polarity in epithelia and influence the balance between stem cell renewal and differentiation [35]–[37]. Since the invasion margins of several carcinomas are frequently enriched in the expression of laminins and their corresponding integrin receptors [38]–[40], the laminins are regarded as important autocrine factors endorsing tumor progression through their interactions with their receptor counterparts particularly in SCC [41], [42].

In solid tumors, integrin expression patterns display a strong heterogeneity and may vary between different carcinomas, between different tumors of the same type and between different regions of the same tumor [19]. Moreover, different integrins frequently show different expression patterns within a given tumor. For this reason, integrin expression is to be investigated for each type of carcinoma and its existing subtypes individually, and findings ought to be compared to the physiological integrin expression in the corresponding non-malignant epithelial tissue to confine tissue specific alterations. As an initial assessment of previously undetermined integrin expression in esophageal carcinoma, the aim of the present study was to analyze the expression magnitude and distribution of the laminin-binding integrins α_2_β_1_, α_3_β_1_, α_6_β_1_, and α_6_β_4_ in ESCC. In order to address their potential as diagnostic and prognostic immunopathological markers, we correlated the integrin staining results with histopathological tumor parameters and postoperative patient survival.

## Materials and Methods

### Patients

The local ethics committee approved the study and written informed consent was obtained from all patients included in the study. Each of the patients underwent primary esophagectomy at the University Medical Center Hamburg-Eppendorf, Hamburg, Germany, in between April 1992 and December 1999. The tumors were staged and graded by pathologists according to the sixth edition of the TNM-classification recommended by the International Union Against Cancer (UICC) and World Health Organization (WHO). Follow-up data was available from 34 of the 36 patients. Three patients with residual tumors (R1) were excluded from Kaplan-Meier survival analysis as well as one patient due to distant metastasis (M1) and three patients surviving less than one month after surgery. The median follow-up period for the remaining 27 patients was 26 months (range: 2–108 months).

### Tissue sampling

Tissue samples were taken from the surgical specimens immediately after esophagectomy, embedded in Tissue-Tek O.C.T. compound (Sakura Finetek, Zoeterwoude, The Netherlands), and instantly snap-frozen in liquid nitrogen. The samples were stored at −80°C until further processing. Serial 5 µm frozen sections were prepared on a microtome cryostat (Microm International, Walldorf, Germany), attached to positively charged glass slides (Histobond, Paul Marienfeld, Lauda-Königshofen, Germany), and air-dried at room temperature. One consecutive section of each tumor sample was stained with hematoxylin and eosin to assess tissue morphology. The remaining slides were stored at −20°C until immunofluorescence staining was performed.

### Immunofluorescence staining

After fixation in ice-cold 100% acetone for 90 seconds, the sections were rehydrated in phosphate buffered saline (PBS; pH 7.4). Incubation procedures were performed in a humid incubation chamber at room temperature. Subsequent to each step, the sections were rinsed three times in PBS for five minutes. Initially, a protein reagent was added to the sections for 20 minutes to block unspecific bonds minimizing background staining (Protein Block Serum-free, Dako, Hamburg, Germany). After this, consecutive sections of each tumor sample were incubated with anti-human monoclonal antibodies against the integrin subunits α_2_ (AK-7), α_3_ (C3 II.1), α_6_ (GoH3), β_1_ (MAR4), and β_4_ (450-9D), respectively (all primary antibodies were purchased from BD Pharmingen, San Diego, CA). The primary antibodies were diluted 1∶200 in a ready-made buffer solution (Antibody Diluent, Dako, Hamburg, Germany) and applied for 60 minutes. Likewise, two sections of each tumor sample were incubated with antibodies against non-human epitopes and served as primary antibody isotype controls (MOPC21, Mouse Myeloma IgG_1_κ, Sigma-Aldrich, St. Louis, MO; anti-KLH Rat IgG_2a_κ, Pharmingen, San Diego, CA). Tissue sections incubated without primary antibody served as negative controls to address potential autofluorescence of the tissue. To confirm the specificity of antibody binding, frozen sections of normal colonic mucosa expressing the analyzed integrins in well-known distributions were included in each staining run as positive controls. [23], [43]. Afterwards, all sections were incubated with Rhodamine-Red-X (RRX) labeled secondary antibodies (AffiniPure Donkey Anti-Mouse/Anti-Rat IgG (H+L), Jackson ImmunoResearch, West Grove, PA) for 60 minutes. Finally, the sections were counterstained applying a mounting medium that contained 4',6-Diamidino-2-phenylindole (Vectashield Mounting Medium with DAPI, Vector Laboratories, Burlingame, CA).

The stained sections were examined with a Leica DMRXA fluorescence microscope (Leica Microsystems, Wetzlar, Germany). Digital images were captured under standard conditions (wide open aperture; 1,500 msec exposure time; 100fold magnification) with a monochrome CCD-camera (Photometric Sensys, Visitron Systems, Puchheim, Germany) using the Leica QFISH software V2.2 (Leica Microsystems Imaging Solutions, Cambridge, UK). Applying fluorescence filters for the specific visualization of DAPI, RRX, and fluorescein isothiocyanate (FITC) successively, serial exposures of each tissue section were obtained.

### Analysis and evaluation of immunofluorescence staining

*Level* (staining intensities) and *pattern* (distribution) of integrin expression were analyzed separately. The Leica QFISH software permits the non-dimensional measurement of luminance raw intensities in digitalized CCD-images of fluorescence labeled structures. We adopted this function for the objective quantification of fluorescence staining in the processed esophageal and colonic tissue sections measuring staining intensities in at least three low power fields (100fold magnification) representative for the tissue section assessed. Raw intensities for Rhodamine Red-X (RRX) fluorescence in specifically immunostained tissue ranged from below 50 to 1800, while non-specific background staining ranged from below 50 to 110. Observing tissue-specific distributions of raw intensity values particularly in ESCC primary tumors, we determined the full range of raw intensities measured in designated areas of the tissue samples. The resulting mean intensity values were classified according to an established semiquantitative scoring system comprising the following *levels* of expression: If mean raw intensities were below 150, non-expression (−) was postulated. Expression was defined as weak (+), if mean staining intensities ranged from 150 to 500. If raw intensities were averaging from 500 to 1000, expression was termed as moderate (++), and if mean raw intensities exceeded 1000, a strong expression (+++) was assigned.

**Figure 1:**
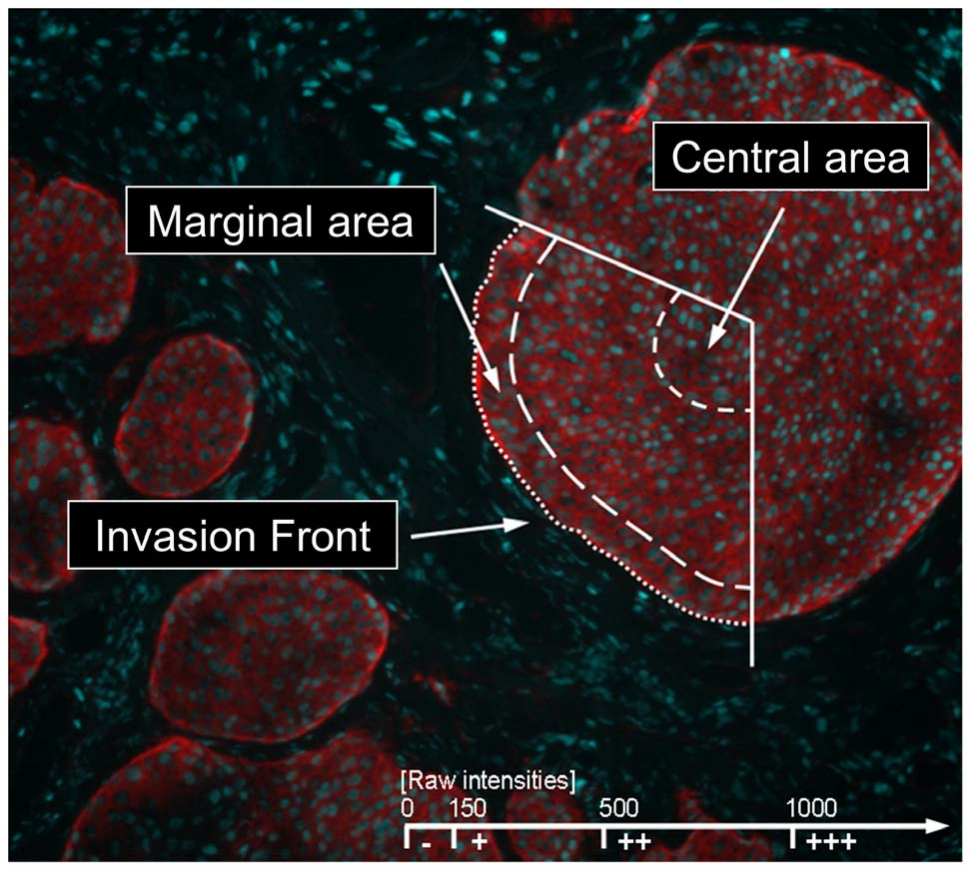
Non-dimensional raw intensities for Rhodamine Red-X (RRX) fluorescence were measured in at least three low power fields (100fold magnification) representative for each tissue section in three different areas of the tumor cell formations: Invasion front, marginal areas, and central areas. Predominant raw intensity values were classified below 150 as negative (−), from 150 to 500 as weak (+), from 500 to 1000 as moderate (++), and above 1000 as strong (+++) expression of the respective integrin subunit.

In each digitalized CCD-image of a low power field mean raw intensities were determined separately by scanning the range of luminance values in three different areas of the tumor cell formations: (1) At the direct invasion front (stromal surfaces of basal tumor cells constituting the invasive tumor margin), (2) in the marginal areas (basal cell layers adjacent to the surrounding tissue), and (3) in the central areas of tumor cell formations. In addition, if tumor sections contained adjacent normal esophageal mucosa, distribution and intensity of integrin expression intensities were determined in an analogous manner (1) at the border of the epithelium to the basement mebrane (basal epithelial surface), (2) in the basal cell layers (stratum basale), (3) in the suprabasal cell layers (stratum spinosum), and (4) in the luminal cell layers (stratum squamosum) of the squamous epithelium (Fig. 1). The mean staining intensities of the integrin subunits measured in the stratum basale of the epithelia served as a reference to evaluate the integrin expression in the suprabasal and central tumor tissue. Furthermore, the average staining intensities at the basal surface of the keratinocytes directly attached to the substratum provided the reference to evaluate the integrin expression at the invasion front of the tumors.

Surveying the entire tumor tissue in the sections, we also evaluated the distribution of the integrin subunits (expression *pattern*). For that purpose, the following staining patterns were distinguished: If integrins were uniformly expressed by more than 75% of the analyzed tumor cells, this was defined as a *homogeneous* expression pattern. If tumors showed a gradually diminishing integrin expression from the tumor invasion front to the tumor center, this was defined as a *polarized* expression pattern. If tumors showed a uniform, non-polarized integrin expression, a *diffuse* expression pattern was postulated and further distinguished between a *diffuse homogenous* expression if more than 75% of the tumor cells were positive and a *diffuse heterogeneous* expression if less than 75% of the tumor cells were positive.

The evaluation of both the staining intensities and staining patterns were performed by two of the authors independently (Christian Vay, Peter Scheunemann) without knowledge of histopathological parameters or patient survival outcome.

### Statistical analysis

Associations between categorical parameters were assessed via Fisher's exact test and, whenever appropriate, with the χ^2^-test. The Kaplan-Meier method was used to estimate overall survival, relapse-free, and disease-specific survival. For comparison purposes log-rank tests were performed. Cox's proportional-hazards models were fitted for multivariate analysis. Relative risk and 95% confidence limits are presented. Differences between groups are considered significant if the *p*-values were less than 0.05 for a two-tailed test (software SPSS 16.0, SPSS, Chicago, IL).

Hierarchical cluster analysis of integrin expression parameters was performed using the *Cluster* software (version 2.11) and the *Treeview* software (version 1.60) which is openly accessible at http://rana.lbl.gov/EisenSoftware.htm. The software had been developed to analyze data according to similarity in patterns of expression without being specifically linked to any particular method generating the data [44].

## Results

### Patient characteristics

Table S1 shows the major clinicopathologic characteristics of the study patients.

### Expression of the integrin subunits α_2_, α_3_, α_6_, β_1_, and β_4_ in esophageal squamous epithelium

Samples of normal esophageal mucosa were available from 19 patients (53%). The complete immunostaining results of the integrin subunits α_2_, α_3_, α_6_, β_1_, and β_4_ in the different layers of normal esophageal mucosa (EM) are shown in Table 1. Generally, the strongest integrin expression was observed in the basal keratinocytes, while staining intensities gradually diminished with an increasing distance to the substratum in terms of a *polarized* expression pattern (Supplementary Table S2). Moreover, the staining intensity of the integrin subunits was distinctly increased at the direct interface of the basal keratinocytes to the substratum. This focal enhancement of integrin expression was observed in 100% of the sections stained for α_6_ (19/19) and β_4_ (18/18), in 83% (15/18) of the sections stained for α_3_, and in 44% (7/16) of the sections stained for β_1_, whereas none of the samples showed an enhanced α_2_ expression (0/19).

**Table 1:** Integrin staining scores in esophageal squamous epithelium (*).

Integrin subunit	Esophageal epithelium	Staining scores (%)							
		+		++		+++		−	
**α_2_**	Basal epithelial surface	0	(0)	0	(0)	0		19	(100)
	Stratum basale	17	(89)	2	(11)	0		0	
	Stratum spinosum	19	(100)	0		0		0	
	Stratum squamosum	6	(32)	0		0		13	(68)
**α_3_**	Basal epithelial surface	12	(66)	3	(17)	0		3	(17)
	Stratum basale	15	(83)	2	(11)	0		1	(6)
	Stratum spinosum	15	(83)	0		0		3	(17)
	Stratum squamosum	2	(11)	0		0		16	(89)
**α_6_**	Basal epithelial surface	1	(5)	5	(26)	13	(69)	0	
	Stratum basale	14	(74)	4	(21)	1	(5)	0	
	Stratum spinosum	17	(90)	1	(5)	1	(5)	0	
	Stratum squamosum	1	(5)	1	(5)	0	(0)	17	(90)
**β_1_**	Basal epithelial surface	6	(38)	1	(6)	0		9	(56)
	Stratum basale	14	(88)	1	(6)	0		1	(6)
	Stratum spinosum	15	(94)	0		0		1	(6)
	Stratum squamosum	2	(12)	0		0		14	(88)
**β_4_**	Basal epithelial surface	10	(56)	8	(44)	0		0	
	Stratum basale	14	(78)	0		0		4	(22)
	Stratum spinosum	8	(44)	0		0		10	(56)
	Stratum squamosum	0		0		0		18	(100)

* For the integrin subunits α_6_, α_3_, β_1_, and β_4_ 19, 18, 16, and 18 cases were evaluated, respectively.

### Expression of the integrin subunits α_2_, α_3_, α_6_, β_1_, and β_4_ at the tumor invasion front

**Figure 2:**
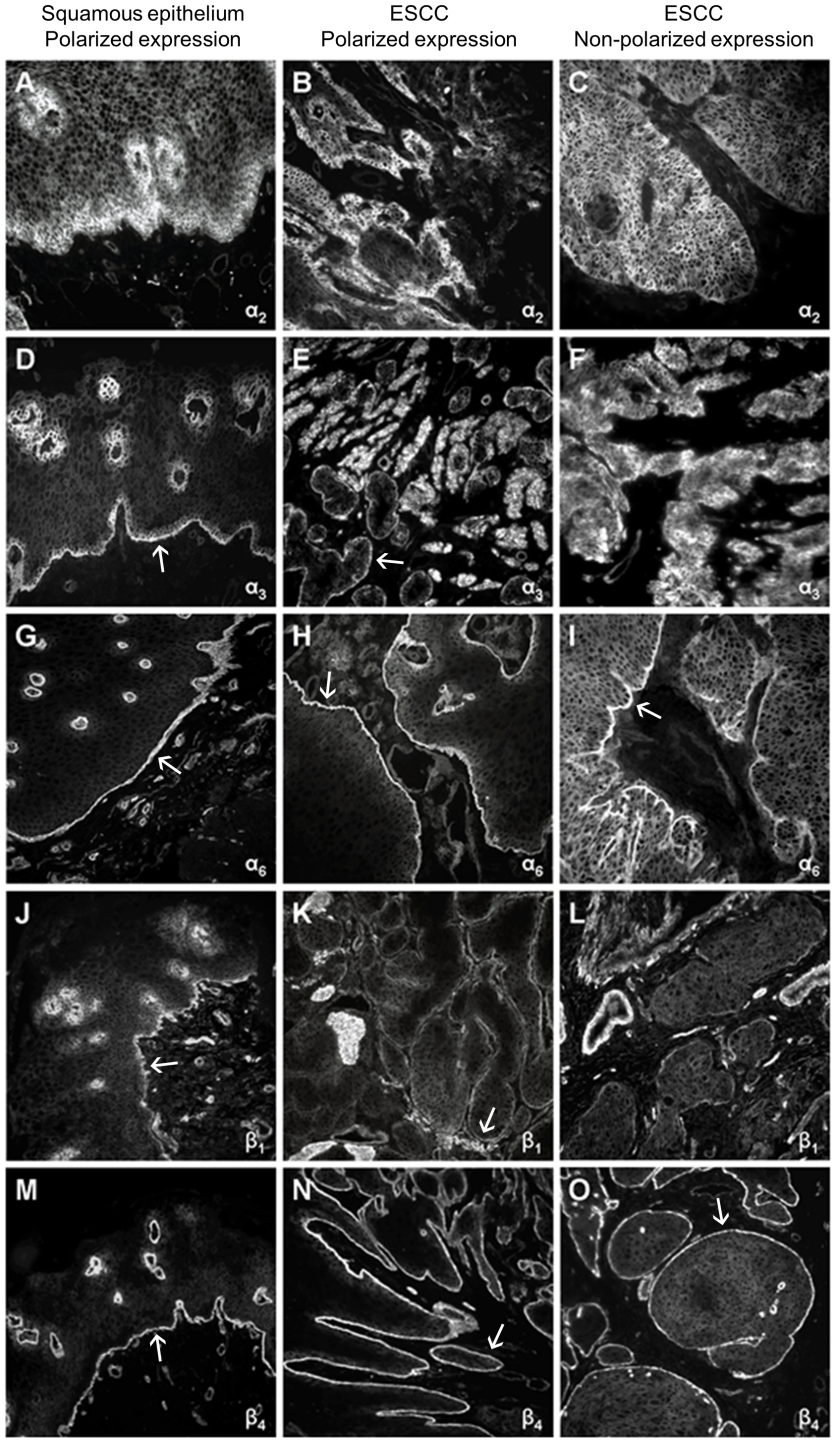
Immunofluorescence staining patterns of the integrin subunits α_2_, α_3_, α_6_, β_1_, and β_4_ in tissue sections of normal esophageal squamous epithelium and esophageal squamous cell carcinoma (ESCC). Magnification 100 fold. Enhanced staining intensities at the basal surface of the epithelium and at the tumor invasion front are marked by an arrow (↑).

In all cases with an enhanced integrin expression at the tumor invasion front, the staining intensities were measurably increased in comparison to the marginal and central tumor areas (Fig. 2 and Table 2). This *distinct amplification of* expression was observed for the α_6_ and the β_4_ subunit in 97% (35/36) and 94% (32/43) of the tumors, respectively. In contrast, a clearly enhanced α_2_, α_3_, and β_1_ expression at the tumor invasion front was observed in only 8% (3/36), 25% (9/36), and 30% (9/30) of the tumors, respectively.

**Table 2:** Integrin staining scores in primary ESCC (n = 36) (*).

Integrin	Primary tumor	Staining scores (%)							
subunit	cell formations	+		++		+++		−	
**α_2_**	Invasion front	28	(78)	5	(14)	2	(5)	1	(3)
	Marginal areas	28	(78)	5	(14)	2	(5)	1	(3)
	Central areas	29	(81)	4	(11)	2	(5)	1	(3)
**α_3_**	Invasion front	23	(64)	11	(30)	1	(3)	1	(3)
	Marginal areas	25	(69)	9	(25)	1	(3)	1	(3)
	Central areas	26	(72)	5	(14)	0		5	(14)
**α_6_**	Invasion front	5	(14)	12	(33)	19	(53)	0	
	Marginal areas	20	(56)	12	(33)	4	(11)	0	
	Central areas	24	(67)	9	(25)	3	(8)	0	
**β_1_**	Invasion front	21	(70)	7	(24)	1	(3)	1	(3)
	Marginal areas	21	(70)	7	(24)	1	(3)	1	(3)
	Central areas	22	(73)	5	(17)	1	(3)	2	(7)
**β_4_**	Invasion front	22	(65)	11	(32)	0		1	(3)
	Marginal areas	26	(76)	0		0		8	(24)
	Central areas	23	(68)	0		0		11	(32)

* The evaluation of the integrin subunits β_1_ and β_4_ was limited to 30 and 34 cases, respectively.

Correlation of immunostaining results with histopathologic tumor characteristics revealed that down-regulation of the α_6_ integrin expression at the tumor invasion front, compared to the generally strong α_6_ expression (+++) along the basement membrane in normal EM (as shown above), was associated with a poor histopathologic tumor grading (G3). While 11 (39%) of the 28 well to moderately differentiated tumors (G1–2) tumors exhibited a decreased α_6_ expression, six (75%) of the eight tumors with a poor differentiation (G3) showed a weak or moderate expression (+/++) at the stromal surface of the basal tumor cells (p = 0.083).

In addition, there was a significant correlation between the enhanced expression of β_1_ at the tumor invasion front and the absence of regional lymph node metastasis. Seven (54%) of the 13 pN0 patients had an enhanced β_1_ expression at the invasion front of their tumors compared to only two (12%) of 17 of the pN1 patients (p = 0.018).

**Figure 3:**
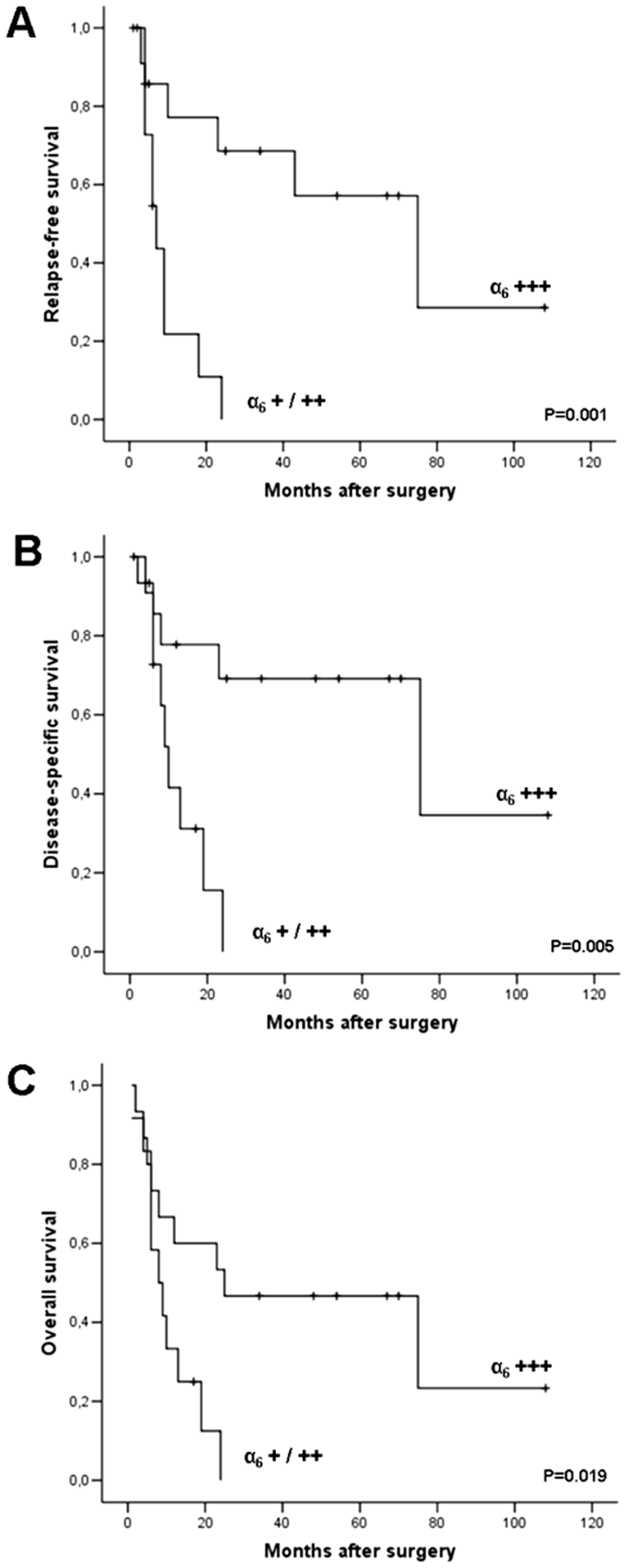
Kaplan-Meier survival analysis. Patients (n = 27) were grouped according to the staining scores of the integrin subunit α_6_ at the invasion front of their primary tumors (12 (α_6_ +++) patients vs. 15 (α_6_ +/++) patients). P values were calculated with the log-rank test (Mantel-Cox). (A) Overall survival. (B) Disease-specific survival. (C) Relapse-free survival.

Kaplan-Meier survival analysis revealed that a strong (+++) α_6_ expression at the invasion front of the tumor was positively correlated with a significantly prolonged postoperative survival (Fig. 3 and Supplementary Table S3). The median relapse-free survival of 15 patients whose tumors showed a strong staining of the α_6_ subunit at the invasion front was 75 months compared to 12 patients with a low or moderate α_6_ staining (+/++) whose median survival was 7 months (p = 0.001). The disease-specific survival and the overall survival of the 15 patients with strong α_6_ staining (+++) at the invasive tumor front was 75 months and 25 months when compared to the 12 patients with a low or moderate α_6_ staining (+/++) whose median survival was 10 months and 8 months, respectively (p = 0.005; p = 0.019).

Moreover, multivariate Cox regression analysis (Table 3) confirmed an independent prognostic influence of the α_6_ expression for relapse-free survival (p = 0.003), tumor-specific survival (p = 0.010), and overall survival (p = 0.028).

**Table 3:** Multivariate survival analysis for relapse-free, disease-specific survival, and overall survival (*).

Parameter	RR	95% CI	p-Value
**Relapse-free survival**			
*Depth infiltration of the primary tumor (pT)*			
pT3–4 vs. pT1–2	1.36	0.46–4.04	0.657
*Regional lymph nodes (pN)*			
pN1 *vs.* pN0	1.63	0.48–5.56	0.434
*Grading (G)*			
G3 vs. G1–2	0.99	0.22–4.42	0.989
*α_6_ integrin staining at the invasion front*			
Down-regulation (+/++) vs. normal expression (+++)	6.41	1.88–21.78	0.003
**Tumor-specific survival**			
*Depth infiltration of the primary tumor (pT)*			
pT3–4 vs. pT1–2	2.15	0.63–7.31	0.220
*Regional lymph nodes (pN)*			
pN1 *vs.* pN0	1.52	0.34–6.87	0.585
*Grading (G)*			
G3 vs. G1–2	0.95	0.18–4.80	0.953
*α_6_ integrin staining at the invasion front*			
Down-regulation (+/++) vs. normal expression (+++)	5.11	1.47–17.81	0.010
**Overall survival**			
*Depth infiltration of the primary tumor (pT)*			
pT3–4 vs. pT1–2	1.75	0.65–4.70	0.271
*Regional lymph nodes (pN)*			
pN1 *vs.* pN0	1.23	0.41–3.67	0.717
*Grading (G)*			
G3 vs. G1–2	0.81	0.22–2.97	0.747
*α_6_ integrin staining at the invasion front*			
Down-regulation (+/++) vs. normal expression (+++)	3.04	1.13–8.21	0.028

* RR  =  relative risk for death; CI  =  confidence interval. Univariate analysis was performed by Kaplan-Meier method and log-rank test (Mantel-Cox).

Thereby, patients with a down-regulated low to moderate (+/++) α_6_ immunostaining at the invasion front shared a 6.41 times increased risk for tumor relapse (95% CI: 1.88–21.78), a 5.11 times increased risk for shortened tumor-associated survival (95% CI: 1.47–17.81), and a 3.04 times increased risk for shortened overall survival (95% CI: 1.13–8.21) compared to patients with a distinct strong (+++) α_6_ staining.

Comparing the survival analyses of overall α_6_ down-regulation at the invasion front to the corresponding expression of β_4_ (Supplementary Fig. S1), the Kaplan-Meier curves exhibited similar shapes and resembled each other in direction. This observation was not applicable to any other combination among the assessed integrin subunits.

### Expression of the integrin subunits α_2_, α_3_, α_6_, β_1_, and β_4_ in suprabasal and central tumor areas

Integrin staining scores (staining intensity and expression pattern) were determined in the marginal and central areas of 36 tumors for the integrin subunits α_2_, α_3_, and α_6_, in 30 tumors for β_1_, and in 34 tumors for β_4_ (Table 2).

An overexpression was assumed if the staining intensity was higher and a down-regulation of integrin expression was supposed if the staining intensity was lower in the suprabasal tumor cells in comparison to the median level of integrin expression in normal suprabasal esophageal epithelium (Table 1; stratum spinosum). Accordingly, integrin overexpression in the tumor tissue was observed in 19% (7/36) of the tumors stained for α_2_, in 28% (10/36) of the tumors stained for α_3_, in 44% (16/36) of the tumors stained for α_6_, and in 27% (8/30) of the tumors stained for β_1_. β_4_ overexpression was not detected (0/34). In contrast, down-regulation was only observed in single tumors stained for the integrin α_2_ (1/36), α_3_ (1/36), and β_1_ chain (1/30), respectively. It was absent for the β_4_ subunit due to the lack of β_4_ integrin expression in the suprabasal normal esophageal epithelium.

We statistically investigated the association between integrin overexpression and histopathological findings. Fisher's exact test revealed that an overexpression of the integrin subunit α_2_ in the suprabasal tumor areas occurred significantly more frequently in patients with pT3-4 tumors compared to patients with pT1-2 tumors (p = 0.041). Kaplan-Meier analysis did not reveal any significant correlation between overexpression and patient survival.

### Expression patterns of the integrin subunits α_2_, α_3_, α_6_, β_1_, and β_4_ in ESCC

Assessing the distribution of the integrin subunits in the tumor sections, *polarized* expression patterns analogous to the integrin distribution in normal esophageal epithelium were distinguished from *diffuse homogeneous* or otherwise *heterogeneous* staining in the tumor tissue (Fig. 2).

Polarized expression was observed in 14% (5/36) of the sections stained for the α_2_ chain, 39% (14/36) of the sections stained for the α_3_ chain, 28% (10/36) stained for the α_6_ chain, 20% (6/30) stained for the β_1_ chain, and 26% (9/34) stained for β_4_. Diffuse homogeneous expression was found in 25% (9/36) of the sections analyzed for the distribution of the α_2_ subunit, 11% (4/36) analyzed for α_3_, 33% (12/36) analyzed for α_6_, 33% (10/30) analyzed for β_1_, and 44% (15/34) analyzed for β_4_. In the remaining cases, the organized expression of the respective integrin subunits was lost and designated as a heterogeneous expression.

We statistically analyzed the association between integrin staining patterns and histopathological findings and performed Kaplan-Meier survival analysis. Polarized expression of the integrin subunits α_6_, β_1_, and β_4_ significantly correlated with prolonged relapse-free patient survival (p = 0.028, p = 0.034, p = 0.006) and was associated with prolonged disease-specific patient survival (p = 0.067, p = 0.014, p = 0.021). Furthermore, polarized expression of the β_1_ integrin subunit significantly correlated with overall survival (p = 0.013) and with the absence (pN0) of regional lymph node metastasis (p = 0.040). Polarized expression of the α_6_ chain was also associated to a limited number (n≤3) of regional lymph node metastases (p = 0.033), whereupon the detection of maximally three lymph node metastases significantly correlated to a prolonged relapse-free survival (p = 0.013). In addition, polarized staining for the β_4_ subunit significantly correlated with the absence of tumor relapse (p = 0.006).

The maintenance of a polarized expression pattern was not necessarily associated to a focally enhanced integrin expression at the stromal surface of the tumors. However, polarized α_6_ and β_4_ expression correlated significantly to each other (p = 0.001). Moreover, we found that polarized staining patterns for the α_6_ and β_4_ subunit both significantly correlated with a strong α_6_ immunoreactivity (+++) at the tumor invasion front (p = 0.047; p = 0.014).

### Hierarchical cluster analysis of integrin expression profiles

**Figure 4:**
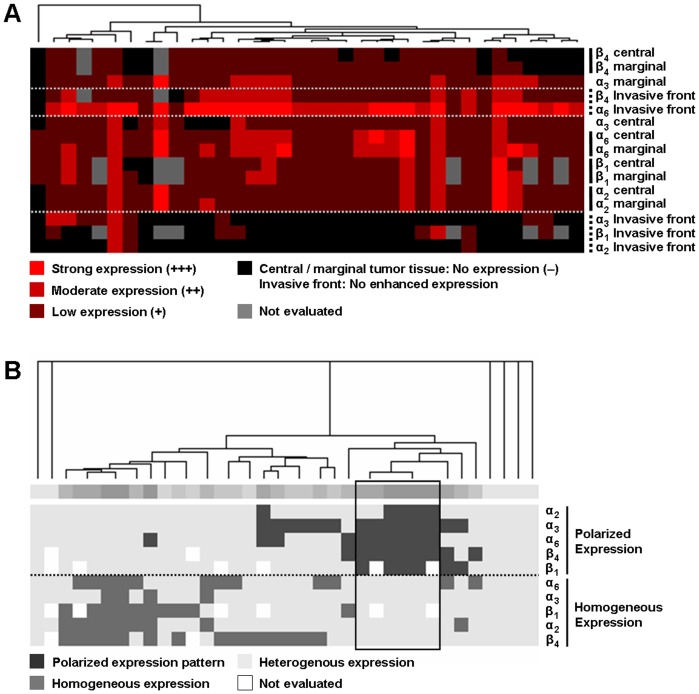
Clustered display of integrin expression characteristics. Each tumor sample is represented by a single column of boxes; each integrin expression parameter is represented by a single row. The samples (columns) are grouped according to the similarity of their expression characteristics. The dendrogram above the cluster encloses subsets of samples reflecting the particular similarity among each other. The profiles of the various expression parameters (rows) are also arranged to each other according to their similarity. (A) Clustered display of the staining scores reflecting the expression quantities for the integrin subunits α_2_, α_3_, α_6_, β_1_, and β_4_ in distinct areas of the tumor samples (invasion front, marginal area, central area). In the majority of the cases, a uniform staining score was present in the marginal and central area of the individual tumor sample, which was reflected by the neighboring arrangement of the corresponding expression profiles. Notably, the expression profiles of the integrin subunits α_6_ and β_4_ at the invasion front were grouped adjacent to each other. (B) Clustered display of staining patterns representing the distribution of the integrin subunits α_2_, α_3_, α_6_, β_1_, and β_4_ in the tumor tissue (polarized, homogeneous and heterogeneous staining pattern). A subset of six patients possessed a polarized staining pattern for the integrin subunits α_3_, α_6_, and β_4_ at minimum (framed columns). The tumors of five of the six patients were well to moderately differentiated (G1-2), whereas only one patient had a poorly differentiated tumor (G3). The six patients showed a strong expression (+++) of the α_6_ subunit at the invasion front of their tumors which was statistically identified as the only independent prognostic factor for the collective.

Subjecting the integrin expression patterns to cluster analysis (Fig. 4), tumors with predominantly polarized expression of the integrin subunits could be clearly distinguished from tumors with mainly homogeneous or heterogeneous distribution.

In the majority of the cases, a uniform staining score was present in the marginal and central area of the individual tumor sample, which was reflected by the neighboring arrangement of the corresponding expression profiles. Notably, the expression profiles of the integrin subunits α_6_ and β_4_ at the invasion front were grouped adjacent to each other (Fig. 4A). The subgroups of patients configured as indicated by the dendrogram did not present common histopathological or prognostic findings in comparison to the respective remainder of the collective. Four major subgroups could be discriminated as indicated by the dendrogram (Fig. 4B): (1) Patients whose tumors mainly showed homogeneous expression patterns for the majority of the integrin subunits, (2) patients whose tumors maintained mostly polarized expression patterns, (3) patients whose tumors featured varying expression patterns, and (4) patients whose tumors exhibited a fully heterogeneous expression behavior. A subset of six patients possessed a polarized staining pattern for the integrin subunits α_3_, α_6_, and β_4_ at minimum (framed columns). The tumors of five of the six patients were well to moderately differentiated (G1-2), whereas only one patient had a poorly differentiated tumor (G3). The six patients showed a strong expression (+++) of the α_6_ subunit at the invasion front of their tumors which was statistically identified as the only independent prognostic factor for the collective. After exclusion of one patient with a residual tumor (R1) and one patient who died within 30 days after surgery, Kaplan-Meier analysis revealed a prolonged relapse-free survival interval for the remaining members of the subgroup in comparison to the rest of the patients (p = 0.041).

## Discussion

Using a novel immunofluorescence staining approach, we have investigated a panel of esophageal squamous cell carcinomas (ESCC) and evaluated both the level of expression and the distribution of the subunits constituting the integrins α_2_β_1_, α_3_β_1_, α_6_β_1_, and α_6_β_4_. By the adoption of available FISH software for the digital analysis of immunofluorescence labelled structures, we demonstrated that direct measuring of luminance in stained tissue sections is feasible and, technically, allows an objective high-resolution determination of in-situ protein expression levels.

Analyzing the integrin expression in both the pathological and physiological state by the same method, we have provided a substantiated evaluation of integrin expression in ESCC. Our results indicate that the abrogation of normal integrin expression characteristics, as they are observed in non-malignant squamous epithelium of the esophagus, is a frequent event in esophageal squamous cell carcinoma (ESCC) associated to an unfavorable disease outcome. Reciprocally, we observed that the maintenance of a polarized integrin expression pattern in the primary tumor tissue, which resembles the physiological integrin expression in normal esophageal mucosa, points towards a less aggressive tumor type.

Whereas the majority of the primary tumors showed a predominantly homogeneous or heterogeneous expression pattern of the integrin subunits, we found a subset of patients whose primary tumors reproduced the polarized integrin expression of the epithelium with the strongest expression on the stromal surface of the tumor cell formations. The maintenance of a polarized expression pattern in the tumors was associated with prolonged relapse-free survival of the patients, which was statistically significant regarding the integrin subunits α_6_, β_1_, and β_4_. Moreover, the majority of these tumors was well to moderately differentiated (G1-2) and shared a strong expression of the α_6_ subunit along the invasion front as it were observed at the basal surface of normal esophageal epithelium. Strong expression of the α_6_ subunit at the invasion front of the tumors significantly correlated with relapse-free, disease-free, and overall patient survival arising as the single independent prognostic factor in the present study. Thus, the maintenance of the polarized distribution of the integrin subunits in the tumor tissue appears to reflect a higher level of differentiation and a less aggressive phenotype compared to tumors with aberrant expression patterns. Accordingly, the abrogation of the physiological integrin expression pattern seems to reflect the escape of invasive tumor cells from the parental tissue's tight control of proliferation and differentiation immanent to normal esophageal epithelium.

The loss of spatial organization in integrin expression is a consistent observation in solid tumors, and observations congruent to our findings have been reported for several other squamous cell carcinomas (SCC) [19]. *In vitro* studies have demonstrated that formations of differentiated and non-differentiated colon carcinoma cells do not so much differ in the magnitude of expression but rather in the distribution of the integrins [20]. Differentiated cells exhibited a polarized integrin expression with the strongest expression at the margin of the tumor cell formations, whereas non-differentiated tumor cells did not show any orderly expression. *In vivo*, integrin expression is frequently completely lost in advanced colonic adenocarcinoma [22], whereas in squamous cell carcinoma not a total loss of integrin expression but the abrogation of the physiological expression pattern appears to be the predominant alteration [18].

In SCC of the oral cavity, Watt et al. distinguished three patterns of integrin expression: “Normal” expression, i.e. the integrin expression was confined to the basal layer of neoplastic cells adjacent to the tumor stroma, was discriminated from “overexpression” if integrin expression was found throughout the tumor tissue. In addition, mainly focal but occasionally extensive “loss” of expression was observed [45], [46]. Several studies report that the expression of the α_6_β_4_ integrin throughout the tumor tissue correlated with poor prognosis [38], [47], and that the focal loss of the α_6_β_4_ integrin or the β_1_ integrins was a characteristic of poorly differentiated tumors [48]. Suprabasal expression of α_6_β_4_ in epidermal lesions resulted in an enhanced tumorigenesis [49]. In oral SCC, the loss or dissociation of the integrin α_6_β_4_ was associated with a breakup of the basement membrane and, therefore, could be related to an increased risk of metastasis [50]. In another study, the loss of polarized α_6_β_4_ expression was suggested as a potential early marker of malignancy in oral SCC [51]. Furthermore, Eriksen et al. suggested that the loss of the α_6_β_4_ integrin predicted the risk of lymph node metastasis in SCC of the neck and head at the time of diagnosis [52]. Rabinovitz and Mercurio also stated a relationship between the abrogation of polarized expression of the α_6_β_4_ integrin and the level of malignancy in SCC, calling α_6_β_4_ a “structural and functional anomaly” among the integrins [38], [53], [54].

Though a tumor phenotype may comprise several alterations in its integrin expression profile, the present data suggests a prominent role for α_6_ integrins. Recently, Kwon et al. have presented a comprehensive study targeting the regulation and function of the α_6_ integrins [55]. In their *in vitro* experiments, the authors convincingly show that the molecular interference and down-regulation of α_6_ integrin expression in ESCC cell lines decreases cell proliferation and invasiveness. Moreover, they found supporting functional evidence that the α_6_β_4_ integrin complex plays a leading role in the control of ESCC cell survival as this has been shown for other epithelial malignancies before. Comparing tumor and normal tissue *in vivo*, the authors described an averaged α_6_ integrin overexpression on the mRNA and protein level. However, the authors did not extend their study to the investigation of spacial and histomorphologic characteristics of integrin expression in ESCC as it have been investigated by our present study.

The prognostic benefit for patients with a strong α_6_ expression at the invasion front of their primary tumors draws the attention towards the integrative effects of the α_6_β_1_ and α_6_β_4_ integrins, respectively. In esophageal epithelium, both α_6_ integrins appear to be largely confined to the basal layers in esophageal squamous epithelium [20]. To distinguish α_6_β_4_ from otherwise α_6_β_1_ expression, we performed a hierarchical cluster analysis. According to their similarity, the expression profiles of the integrin subunits α_6_ and β_4_ along the tumor invasion front were grouped adjacent to each other reflecting a high degree of coherence. In addition, the Kaplan-Meier analyses comparing the distinct α_6_ and β_4_ expression at the invasion front exhibited similar curve shapes like no other combination of integrin subunits did. On this account and with regard to the current state of knowledge concerning its expression and function, we deduced that the α_6_β_4_ integrin is responsible for the significant prognostic impact of α_6_ expression at the tumor invasion front of ESCC.

As an integral element of hemidesmosomes, the integrin α_6_β_4_ links intracellular intermediate filaments to the extracellular matrix component laminin and, thereby, anchors the basal epithelial cell layer to the basement membrane [56], [57]. Apart from its mechanistic function, α_6_β_4_ is involved in the regulation of signaling pathways that control actin dynamics and cell movement [53]. In epithelial cells, the release of α_6_β_4_ from disrupting hemidesmosomes unveils its signaling competences, and its association with growth factor receptors as EGFR, HER2, RON, and MET activate Ras- and PI3K-dependent pathways promoting invasion and cell migration [58]–[62].

In contrast to previous assumptions, in which integrins and ErbB receptor tyrosine kinases independently activate downstream signaling upon their specific ligand activation, the research group of Takada et al. have recently demonstrated that – in assembling a ternary complex – a direct binding between neuregulin-1 (NRG1) and the α_6_β_4_ integrin mediates integrin-ErbB crosstalk [63]. Furthermore, the same group described that insulin-like growth factor 1 (IGF1) directly interacts with integrins and that this interaction is required for IGF1 receptor activation [64]. The disruption of such autocrine loops, e.g. by antibody therapy against direct binding sites, represents a promising additional target to restrain cancer cell growth.

In concordance with these findings, the loss of the orderly α_6_ expression along the invasion front would compromise a persistent integrity of tumor cell formations and – releasing α_6_β_4_ to liberate its signaling activities – facilitate the invasion, migration and dissemination of neoplastic cells. Conversely, a sustained strong expression of α_6_ integrins at the stromal tumor surface might indicate a condition closer to the physiological situation in normal esophageal epithelium. In this, state α_6_β_1_ and α_6_β_4_ might not only provide stable attachment to the ECM, but also safeguard cell turnover and tissue homeostasis and, beyond, could be unresponsive to binding-site-specific therapies.

With a view to the loss of growth control as a fundamental step during tumorigenesis and tumor progression, the investigative focus turns to potential “cancer initiating cells” holding dysregulated stem cell properties [65]. Side populations of murine epithelial cells with the capacity for self-renewal and differentiation have been isolated based on their expression of the integrin subunits α_6_ and β_1_ [66]–[68]. In a stem cell model for the esophageal epithelium proposed by Seery and Watt, putative esophageal stem cells, their progenies, and terminally differentiating keratinocytes reside in distinct anatomical regions [37]. Whereas transit-amplifying cells were assigned mainly to epibasal layers, self-renewing keratinocytes with stem cell characteristics are supposed to be strictly confined to the basal cell layer. Based on their findings, the authors suggest that the direct, integrin-mediated contact to the basement membrane safeguards the functional characteristics of esophageal stem cells. While α_6_β_4_ expression along the basal surface of esophageal epithelium was constant, areas with high focal β_1_ expression harbored less putative stem cells in comparison to areas with low β_1_ expression indicating a finely tuned spatial distribution of stem cells. Though we did not measure such variations at the basal aspect of individual epithelia and/or tumor cell formations in our study, the strength of integrin expression at the invasion front varied significantly between different tumors and could mirror the aberrant proliferative, invasive, and, eventually, metastatic behavior of the primary tumor cells.

Even though our study comprised a limited number of cases, the results comprehensively describe alterations in both magnitude and pattern of integrin expression in esophageal squamous cell carcinoma that strongly encourage further investigation. Particularly the expression of the two α_6_ integrins α_6_β_1_ and α_6_β_4_ appear to play a critical role in the malignant progression of ESCC reflecting its aggressiveness: The abrogation of a polarized expression pattern in the primary tumor with a loss of the focally enhanced integrin expression along the tumor invasion front represents an amendatory histopathological marker to further assess the malignancy of the individual tumor. Subsequent studies with greater case numbers incorporating esophageal adenocarcinoma as well as lymph node and distant metastases shall extend our understanding of the integrins' role in the progression of esophageal cancer.

## Supporting Information

Figure S1 **Correlating the survival analyses for integrin α_6_ down-regulation at the invasion front in disease-specific, relapse-free, and overall survival to the corresponding expression of the β_4_ subunit, the Kaplan-Meier calculations exhibited rectified and equaling curves.**
(TIF)

Table S1 Patient and tumor characteristics.
(DOC)

Table S2 Integrin staining scores in esophageal squamous epithelium.
(DOC)

Table S3 Univariate analysis for relapse-free, disease-specific, and overall survival.
(DOC)
