# Supplementary material for: Integrin Expression in Esophageal Squamous Cell Carcinoma: Loss of the Physiological Integrin Expression Pattern Correlates with Disease Progression
Source: PLoS One. 2014 Nov 14;9(11):e109026. doi: 10.1371/journal.pone.0109026 (PMC4232252; doi:10.1371/journal.pone.0109026)
Supplement: Table S1 — Patient and tumor characteristics. (DOC) [file pone.0109026.s002.doc]

Table S1. Patient and tumor characteristics.

| **Parameter** | | **No. of patients (%)** | |
| --- | --- | --- | --- |
| **Patients** | | 36 | (100) |
|  | Male | 30 | (83) |
|  | Female | 6 | (17) |
| **Depth infiltration of the primary tumor (T)** | |  |  |
|  | Tumor invades lamina propria or submucosa (pT1) | 3 | (8) |
|  | Tumor invades muscularis propria (pT2) | 9 | (25) |
|  | Tumor invades adventitia (pT3) | 22 | (61) |
|  | Tumor invades adjacent structures (pT4) | 2 | (6) |
| **Regional lymph nodes (N)** | |  |  |
|  | No regional lymph node metastasis (pN0) | 14 | (39) |
|  | Regional lymph node metastasis (pN1) | 22 | (61) |
| **Number of regional lymph node metastases (pLN)** | |  |  |
|  | Three or less regional lymph node metastases (pLN ≤ 3) | 27 | (75) |
|  | More than three regional lymph node metastases (pLN > 3) | 9 | (25) |
| **Distant metastasis (M)** | |  |  |
|  | No distant metastasis (M0) | 33 | (92) |
|  | Distant lymph node metastasis (pM1a) | 2 | (6) |
|  | Other distant metastasis (pM1b) | 1 | (3) |
| **UICC classification** | |  |  |
|  | Stage I | 2 | (6) |
|  | Stage IIa | 12 | (33) |
|  | Stage IIb | 5 | (14) |
|  | Stage III | 14 | (39) |
|  | Stage IVa | 2 | (6) |
|  | Stage IVb | 1 | (2) |
| **Histologic grade (G)** | |  |  |
|  | Well differentiated (G1) | 1 | (3) |
|  | Moderately differentiated (G2) | 26 | (72) |
|  | Poorly differentiated (G3) | 9 | (25) |
