# Supplementary material for: Integrin Expression in Esophageal Squamous Cell Carcinoma: Loss of the Physiological Integrin Expression Pattern Correlates with Disease Progression
Source: PLoS One. 2014 Nov 14;9(11):e109026. doi: 10.1371/journal.pone.0109026 (PMC4232252; doi:10.1371/journal.pone.0109026)
Supplement: Table S2 — Integrin staining scores in esophageal squamous epithelium. (DOC) [file pone.0109026.s003.doc]

Table S2. Integrin staining scores in esophageal squamous epithelium (*).

| **Integrin subunit** | **Basal epithelial surface** | | **Stratum** | | | | | |
| --- | --- | --- | --- | --- | --- | --- | --- | --- |
|  |  |  | **basale** | | **spinosum** | | **squamosum** | |
| **α_2_** | - | (100) | ++ / + | (100) | + | (100) | + / - | (100) |
| **α_3_** | ++ / + | (83) | ++ / + | (94) | + | (83) | - | (89) |
| **α_6_** | +++ / ++ | (95) | ++ / + | (96) | + | (90) | - | (90) |
| **β_1_** | + / - | (94) | ++ / + | (94) | + | (94) | - | (88) |
| **β_4_** | ++ / + | (100) | + / - | (100) | + / - | (100) | - | (100) |

* Symbols indicate the dominant staining scores in the various epithelial layers (percentages in parentheses).
