# Supplementary material for: Integrin Expression in Esophageal Squamous Cell Carcinoma: Loss of the Physiological Integrin Expression Pattern Correlates with Disease Progression
Source: PLoS One. 2014 Nov 14;9(11):e109026. doi: 10.1371/journal.pone.0109026 (PMC4232252; doi:10.1371/journal.pone.0109026)
Supplement: Table S3 — Univariate analysis for relapse-free, disease-specific, and overall survival. (DOC) [file pone.0109026.s004.doc]

Table S3. Univariate analysis for relapse-free, disease-specific, and overall survival (*).

| **Parameter** | **Months (Median)** | **p-Value** |
| --- | --- | --- |
| **Relapse-free survival** |  |  |
| *Depth infiltration of the primary tumor (pT)* |  |  |
| pT3–4 vs. pT1–2 | 18 vs. 24 | 0.886 |
| *Regional lymph nodes (pN)* |  |  |
| pN1 vs. pN0 | 9 vs. 43 | 0.085 |
| *UICC stage* |  |  |
| UICC III–IV vs. UICC I–II | 9 vs. 43 | 0.025 |
| *Grading (G)* |  |  |
| G3 vs. G1–2 | 7 vs. 23 | 0.172 |
| *No. of lymph node metastases* |  |  |
| n > 3 vs. n ≤ 3 | 7 vs. 43 | 0.013 |
| *α_6_ integrin staining at the invasion front* |  |  |
| Down-regulation (+/++) vs. normal expression (+++) | 7 vs. 75 | 0.001 |
| **Disease-specific survival** |  |  |
| *Depth infiltration of the primary tumor (pT)* |  |  |
| pT3–4 vs. pT1–2 | 19 vs. 24 | 0.516 |
| *Regional lymph nodes (pN)* |  |  |
| pN1 vs. pN0 | 13 vs. 75 | 0.106 |
| *UICC stage* |  |  |
| UICC III–IV vs. UICC I–II | 10 vs. 75 | 0.008 |
| *Grading (G)* |  |  |
| G3 vs. G1–2 | 23 vs. 24 | 0.465 |
| *No. of lymph node metastases* |  |  |
| n > 3 vs. n ≤ 3 | 23 vs. 24 | 0.242 |
| *α_6_ integrin staining at the invasion front* |  |  |
| Down-regulation (+/++) vs. normal expression (+++) | 10 vs. 75 | 0.005 |
| **Overall survival** |  |  |
| *Depth infiltration of the primary tumor (pT)* |  |  |
| pT3–4 vs. pT1–2 | 9 vs. 12 | 0.504 |
| *Regional lymph nodes (pN)* |  |  |
| pN1 vs. pN0 | 10 vs. 23 | 0.250 |
| *UICC stage* |  |  |
| UICC III–IV vs. UICC I–II | 6 vs. 25 | 0.007 |
| *Grading (G)* |  |  |
| G3 vs. G1–2 | 6 vs. 12 | 0.708 |
| *No. of lymph node metastases* |  |  |
| n > 3 vs. n ≤ 3 | 6 vs. 13 | 0.154 |
| *α_6_ integrin staining at the invasion front* |  |  |
| Down-regulation (+/++) vs. normal expression (+++) | 8 vs. 25 | 0.019 |

* Univariate analysis was performed by Kaplan-Meier method and log-rank test (Mantel-Cox).
